# (*E*)-1-(3,5-Difluoro­phen­yl)-3-(2,4-dimeth­oxy­phen­yl)prop-2-en-1-one

**DOI:** 10.1107/S1600536810035257

**Published:** 2010-09-08

**Authors:** Tanxiao Huang, Dongdong Zhang, Quanzhi Yang, Xiaoyan Wei, Jianzhang Wu

**Affiliations:** a School of Pharmacy, Wenzhou Medical College, Wenzhou, Zhejiang Province 325035, People’s Republic of China; b Life Science College, Wenzhou Medical College, Wenzhou, Zhejiang Province 325035, People’s Republic of China; c Institute of Biotechnology, Nanjing University of Science and Technology, Nanjing, Jiangsu Province 210094, People’s Republic of China

## Abstract

The title compound, C_17_H_14_F_2_O_3_, is approximately planar, the dihedral angle between the rings being 5.46 (2)°. The H atoms of the central propenone group are *trans*. The crystal structure is stabilized by inter­molecular C—H⋯F hydrogen bonds.

## Related literature

For related structures, see: Peng *et al.* (2010 ▶); Wu, Zhang *et al.* (2009 ▶); Liang *et al.* (2007 ▶); Yathirajan *et al.* (2006 ▶). For background to and applications of chalcones, see: Nowakowska (2007 ▶); Nielsen *et al.* (2005 ▶); Wu, Qiu *et al.* (2009 ▶); Liang *et al.* (2009 ▶); Mojzisa *et al.* (2008 ▶); Liu *et al.* (2008 ▶); Wu *et al.* (2010 ▶); Zhao *et al.* (2010 ▶); Selvakumar *et al.* (2007 ▶). 
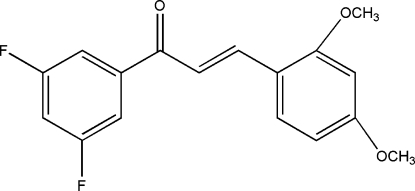


## Experimental

### 

#### Crystal data

C_17_H_14_F_2_O_3_
*M* *_r_* = 304.28
Triclinic, 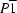

*a* = 7.8047 (8) Å
*b* = 11.2591 (12) Å
*c* = 17.0080 (18) Å
α = 81.407 (2)°
β = 81.231 (2)°
γ = 76.319 (2)°
*V* = 1425.1 (3) Å^3^
*Z* = 4
Mo *K*α radiation
μ = 0.11 mm^−1^
*T* = 293 K
0.27 × 0.22 × 0.17 mm

#### Data collection

Bruker SMART CCD area-detector diffractometer
Absorption correction: multi-scan (*SADABS*; Sheldrick, 1996 ▶) *T*_min_ = 0.602, *T*_max_ = 1.000
7600 measured reflections
5242 independent reflections
2815 reflections with *I* > 2σ(*I*)
*R*_int_ = 0.034

#### Refinement

*R*[*F* ^2^ > 2σ(*F* ^2^)] = 0.057
*wR*(*F* ^2^) = 0.162
*S* = 0.92
5242 reflections
401 parameters
H-atom parameters constrained
Δρ_max_ = 0.24 e Å^−3^
Δρ_min_ = −0.20 e Å^−3^

### 

Data collection: *SMART* (Bruker, 2002 ▶); cell refinement: *SAINT* (Bruker, 2002 ▶); data reduction: *SAINT*; program(s) used to solve structure: *SHELXS97* (Sheldrick, 2008 ▶); program(s) used to refine structure: *SHELXL97* (Sheldrick, 2008 ▶); molecular graphics: *SHELXTL* (Sheldrick, 2008 ▶); software used to prepare material for publication: *SHELXTL*.

## Supplementary Material

Crystal structure: contains datablocks I, global. DOI: 10.1107/S1600536810035257/pb2039sup1.cif

Structure factors: contains datablocks I. DOI: 10.1107/S1600536810035257/pb2039Isup2.hkl

Additional supplementary materials:  crystallographic information; 3D view; checkCIF report

## Figures and Tables

**Table 1:** Hydrogen-bond geometry (Å, °)

*D*—H⋯*A*	*D*—H	H⋯*A*	*D*⋯*A*	*D*—H⋯*A*
C25—H25⋯F4^i^	0.93	2.59	3.375 (3)	142
C13—H13⋯F4^ii^	0.93	2.46	3.303 (4)	151
C9—H9⋯F3^iii^	0.93	2.66	3.532 (3)	156
C30—H30⋯F2^ii^	0.93	2.47	3.303 (4)	149
C8—H8⋯F2^i^	0.93	2.46	3.369 (3)	166
C28—H28⋯F1^iii^	0.93	2.53	3.437 (3)	166

Symmetry codes: (i) 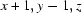; (ii) 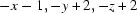; (iii) 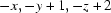.

**Fig. 1.**
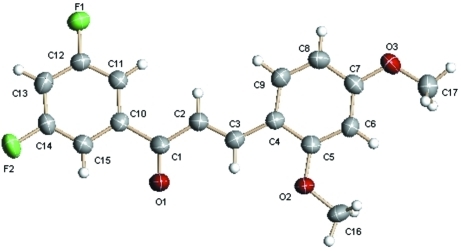
Ellispoid plot.

**Fig. 2.**
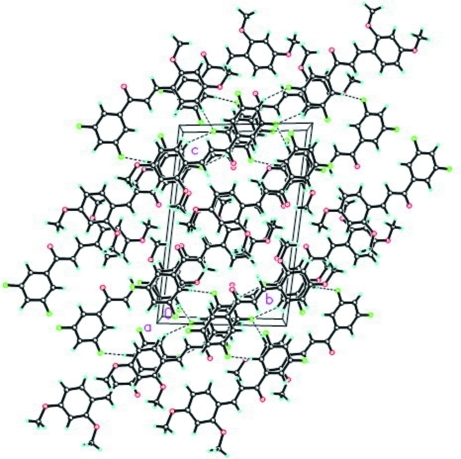
Packing diagram.

## supplementary crystallographic information

### Comment

Chalcones, which have the common Skeleton of 1,3-diaryl-2-propen-1-ones, are open-chain flavonoids. Chalcones belong to nature products and distribute widespread in fruits, vegetables, tea and so on. Like as other flavonoids, Chalcones have wide-range biological properties, including antimicrobial, antitumor, antiangiogenic, antifungal, antioxidant activities and so on (Nowakowska, 2007; Zhao *et al.*, 2010; Liu *et al.*, 2008; Wu *et al.*, 2010.). Moreover, Chalcones have low toxicity. Owing to its good effect and low toxicity, it has attract more and more scientists attention. Recent studies have demonstrated that synthesized Chalcones have the same activities as or better activities than natural chalcones (Nowakowska, 2007; Selvakumar *et al.*, 2007).

Because Chalcones have good activity, the title chalcone derivative has been synthesized. In order to get detailed information such as the geometrical features and the underlying interaction of the crystal structure, an X-ray study of the title compound was carried out.

Two rings of molecule is approximately planar and the dihedral angle between the two rings is 5.46°. The H atoms of the central propenone group are *trans*. The average value of exocyclic bond angles [120.7 (4)°] and the bond distances [1.381 (5) Å] in the phenyl rings are agree quite well with the normal values reported in the literature for some analogous structures (Peng *et al.*, 2010; Wu *et al.*, 2009; Liang *et al.*, 2007; Yathirajan *et al.*, 2006). In the crystal, The crystal structure is stabilized by intermolecular C—H···F hydrogen bonds.

### Experimental

The title compounds was synthesized by Claisene-Schmidt condensation. 2,4-dimethoxyBenzaldehyde (2 mmol) and 3',5'-Difluoroacetophenone (2 mmol) were dissolved in ehanol (20 ml). Temperature of reaction was controlled at 278 K and 5 drops NaOH (20%) was added. The reaction was monitored by thin-layer chromatography. 20 ml H_2_O was added after 8 h and the yellow solid was Precipitated, washed with water and cold ethanol, dried and purified by column chromatography on silica gel. Single crystals of the title compound were grow in a CH_2_Cl_2_/CH_3_CH_2_OH mixture (2:1) at 277 K.

### Crystal data

**Table:**

C_17_H_14_F_2_O_3_	*Z* = 4
*M**_r_* = 304.28	*F*(000) = 632
Triclinic, *P*1	*D*_x_ = 1.418 Mg m^−^^3^
*a* = 7.8047 (8) Å	Mo *K*α radiation, λ = 0.71073 Å
*b* = 11.2591 (12) Å	Cell parameters from 1503 reflections
*c* = 17.0080 (18) Å	θ = 4.7–46.3°
α = 81.407 (2)°	µ = 0.11 mm^−^^1^
β = 81.231 (2)°	*T* = 293 K
γ = 76.319 (2)°	Prismatic, green
*V* = 1425.1 (3) Å^3^	0.27 × 0.22 × 0.17 mm

### Data collection

**Table:**

Bruker SMART CCD area-detector diffractometer	5242 independent reflections
Radiation source: fine-focus sealed tube	2815 reflections with *I* > 2σ(*I*)
graphite	*R*_int_ = 0.034
phi and ω scans	θ_max_ = 25.5°, θ_min_ = 1.9°
Absorption correction: multi-scan (*SADABS*; Sheldrick, 1996)	*h* = −9→9
*T*_min_ = 0.602, *T*_max_ = 1.000	*k* = −6→13
7600 measured reflections	*l* = −20→20

### Refinement

**Table:**

Refinement on *F*^2^	Primary atom site location: structure-invariant direct methods
Least-squares matrix: full	Secondary atom site location: difference Fourier map
*R*[*F*^2^ > 2σ(*F*^2^)] = 0.057	Hydrogen site location: inferred from neighbouring sites
*wR*(*F*^2^) = 0.162	H-atom parameters constrained
*S* = 0.92	*w* = 1/[σ^2^(*F*_o_^2^) + (0.0748*P*)^2^] where *P* = (*F*_o_^2^ + 2*F*_c_^2^)/3
5242 reflections	(Δ/σ)_max_ = 0.005
401 parameters	Δρ_max_ = 0.24 e Å^−^^3^
0 restraints	Δρ_min_ = −0.20 e Å^−^^3^

### Special details

**Table:**

Geometry. All e.s.d.'s (except the e.s.d. in the dihedral angle between two l.s. planes) are estimated using the full covariance matrix. The cell e.s.d.'s are taken into account individually in the estimation of e.s.d.'s in distances, angles and torsion angles; correlations between e.s.d.'s in cell parameters are only used when they are defined by crystal symmetry. An approximate (isotropic) treatment of cell e.s.d.'s is used for estimating e.s.d.'s involving l.s. planes.
Refinement. Refinement of *F*^2^ against ALL reflections. The weighted *R*-factor *wR* and goodness of fit *S* are based on *F*^2^, conventional *R*-factors *R* are based on *F*, with *F* set to zero for negative *F*^2^. The threshold expression of *F*^2^ > σ(*F*^2^) is used only for calculating *R*-factors(gt) *etc*. and is not relevant to the choice of reflections for refinement. *R*-factors based on *F*^2^ are statistically about twice as large as those based on *F*, and *R*- factors based on ALL data will be even larger.

### Fractional atomic coordinates and isotropic or equivalent isotropic displacement parameters (Å^2^)

**Table:**

	*x*	*y*	*z*	*U*_iso_*/*U*_eq_	
F1	−0.1189 (2)	0.41876 (16)	1.15514 (9)	0.0680 (5)	
F2	−0.4957 (2)	0.73654 (16)	1.01469 (11)	0.0774 (6)	
F3	−0.1962 (3)	0.85773 (17)	0.95482 (10)	0.0765 (6)	
F4	−0.5094 (3)	1.19504 (18)	0.80248 (13)	0.1043 (8)	
O1	−0.2308 (3)	0.4469 (2)	0.81479 (12)	0.0796 (8)	
O2	0.0468 (3)	0.10719 (18)	0.67405 (11)	0.0612 (6)	
O3	0.4032 (3)	−0.26852 (18)	0.79562 (12)	0.0638 (6)	
O4	−0.2520 (3)	0.8979 (2)	0.60758 (13)	0.0763 (7)	
O5	0.1805 (3)	0.61876 (18)	0.46058 (11)	0.0595 (6)	
O6	0.4537 (3)	0.21210 (18)	0.57760 (12)	0.0621 (6)	
C1	−0.1733 (4)	0.3926 (3)	0.87639 (17)	0.0503 (8)	
C2	−0.0645 (4)	0.2671 (3)	0.87879 (17)	0.0534 (8)	
H2	−0.0239	0.2287	0.9269	0.064*	
C3	−0.0216 (4)	0.2062 (3)	0.81555 (17)	0.0478 (8)	
H3	−0.0661	0.2473	0.7688	0.057*	
C4	0.0863 (4)	0.0833 (3)	0.81000 (16)	0.0433 (7)	
C5	0.1221 (4)	0.0337 (3)	0.73634 (16)	0.0469 (7)	
C6	0.2271 (4)	−0.0839 (3)	0.72881 (17)	0.0494 (8)	
H6	0.2496	−0.1154	0.6798	0.059*	
C7	0.2975 (4)	−0.1533 (3)	0.79590 (18)	0.0492 (8)	
C8	0.2621 (4)	−0.1065 (3)	0.86926 (18)	0.0580 (9)	
H8	0.3096	−0.1533	0.9139	0.070*	
C9	0.1576 (4)	0.0082 (3)	0.87555 (17)	0.0525 (8)	
H9	0.1329	0.0374	0.9253	0.063*	
C10	−0.2167 (3)	0.4558 (2)	0.95157 (16)	0.0418 (7)	
C11	−0.1461 (4)	0.4034 (3)	1.02204 (16)	0.0457 (7)	
H11	−0.0681	0.3269	1.0249	0.055*	
C12	−0.1941 (4)	0.4671 (3)	1.08689 (16)	0.0473 (7)	
C13	−0.3090 (4)	0.5794 (3)	1.08698 (18)	0.0537 (8)	
H13	−0.3390	0.6210	1.1321	0.064*	
C14	−0.3774 (4)	0.6268 (3)	1.01672 (18)	0.0503 (8)	
C15	−0.3339 (4)	0.5702 (3)	0.94908 (17)	0.0484 (8)	
H15	−0.3814	0.6070	0.9022	0.058*	
C16	0.0892 (5)	0.0699 (3)	0.59549 (16)	0.0703 (10)	
H16A	0.2140	0.0624	0.5787	0.105*	
H16B	0.0229	0.1302	0.5589	0.105*	
H16C	0.0590	−0.0081	0.5960	0.105*	
C17	0.4356 (4)	−0.3267 (3)	0.72383 (19)	0.0662 (9)	
H17A	0.3244	−0.3278	0.7067	0.099*	
H17B	0.5025	−0.4096	0.7340	0.099*	
H17C	0.5018	−0.2817	0.6826	0.099*	
C18	−0.2005 (4)	0.8425 (3)	0.67020 (17)	0.0487 (8)	
C19	−0.0831 (4)	0.7203 (3)	0.67198 (17)	0.0493 (8)	
H19	−0.0649	0.6735	0.7211	0.059*	
C20	−0.0021 (4)	0.6746 (3)	0.60528 (17)	0.0466 (7)	
H20	−0.0241	0.7249	0.5577	0.056*	
C21	0.1172 (4)	0.5552 (2)	0.59737 (16)	0.0423 (7)	
C22	0.2083 (3)	0.5271 (3)	0.52183 (16)	0.0430 (7)	
C23	0.3202 (3)	0.4129 (2)	0.51277 (16)	0.0467 (7)	
H23	0.3787	0.3950	0.4626	0.056*	
C24	0.3438 (4)	0.3257 (3)	0.57915 (17)	0.0465 (7)	
C25	0.2562 (4)	0.3512 (3)	0.65383 (17)	0.0521 (8)	
H25	0.2723	0.2921	0.6981	0.062*	
C26	0.1461 (4)	0.4637 (3)	0.66228 (16)	0.0478 (7)	
H26	0.0885	0.4800	0.7128	0.057*	
C27	−0.2562 (4)	0.9035 (3)	0.74549 (17)	0.0440 (7)	
C28	−0.2002 (4)	0.8479 (3)	0.81899 (17)	0.0496 (8)	
H28	−0.1295	0.7687	0.8238	0.060*	
C29	−0.2513 (4)	0.9119 (3)	0.88340 (17)	0.0500 (8)	
C30	−0.3563 (4)	1.0274 (3)	0.88054 (19)	0.0579 (8)	
H30	−0.3918	1.0683	0.9259	0.070*	
C31	−0.4070 (4)	1.0801 (3)	0.8076 (2)	0.0605 (9)	
C32	−0.3603 (4)	1.0222 (3)	0.74061 (19)	0.0562 (8)	
H32	−0.3975	1.0615	0.6921	0.067*	
C33	0.2640 (4)	0.5973 (3)	0.38187 (16)	0.0594 (9)	
H33A	0.3906	0.5764	0.3817	0.089*	
H33B	0.2316	0.6703	0.3454	0.089*	
H33C	0.2259	0.5307	0.3655	0.089*	
C34	0.5535 (4)	0.1789 (3)	0.50357 (19)	0.0704 (10)	
H34A	0.4738	0.1760	0.4665	0.106*	
H34B	0.6313	0.0996	0.5126	0.106*	
H34C	0.6226	0.2390	0.4818	0.106*	

### Atomic displacement parameters (Å^2^)

**Table:**

	*U*^11^	*U*^22^	*U*^33^	*U*^12^	*U*^13^	*U*^23^
F1	0.0799 (13)	0.0739 (13)	0.0421 (10)	0.0071 (10)	−0.0177 (9)	−0.0090 (9)
F2	0.0863 (14)	0.0563 (12)	0.0775 (13)	0.0256 (10)	−0.0236 (10)	−0.0218 (10)
F3	0.1055 (15)	0.0721 (13)	0.0443 (11)	0.0041 (11)	−0.0221 (10)	−0.0078 (9)
F4	0.139 (2)	0.0618 (13)	0.0955 (16)	0.0442 (13)	−0.0455 (14)	−0.0312 (12)
O1	0.1074 (19)	0.0692 (16)	0.0395 (13)	0.0305 (14)	−0.0166 (12)	−0.0070 (12)
O2	0.0856 (16)	0.0512 (13)	0.0334 (11)	0.0128 (11)	−0.0118 (10)	−0.0025 (10)
O3	0.0758 (15)	0.0462 (13)	0.0605 (14)	0.0126 (11)	−0.0208 (12)	−0.0074 (11)
O4	0.1063 (19)	0.0609 (15)	0.0495 (14)	0.0189 (13)	−0.0280 (13)	−0.0088 (11)
O5	0.0801 (15)	0.0510 (12)	0.0341 (11)	0.0040 (11)	−0.0019 (10)	0.0025 (10)
O6	0.0730 (15)	0.0514 (13)	0.0475 (13)	0.0146 (11)	−0.0073 (11)	−0.0057 (10)
C1	0.0553 (19)	0.0490 (19)	0.0368 (17)	0.0027 (15)	−0.0018 (14)	−0.0004 (14)
C2	0.063 (2)	0.0495 (18)	0.0396 (17)	0.0054 (16)	−0.0091 (14)	−0.0046 (14)
C3	0.0543 (19)	0.0489 (18)	0.0365 (16)	−0.0023 (15)	−0.0075 (13)	−0.0055 (14)
C4	0.0500 (18)	0.0413 (17)	0.0366 (16)	−0.0054 (14)	−0.0070 (13)	−0.0040 (13)
C5	0.0531 (18)	0.0441 (18)	0.0386 (16)	−0.0035 (15)	−0.0053 (13)	−0.0015 (14)
C6	0.0569 (19)	0.0477 (18)	0.0394 (17)	−0.0003 (15)	−0.0050 (14)	−0.0110 (14)
C7	0.0487 (18)	0.0431 (18)	0.0501 (19)	0.0019 (15)	−0.0093 (14)	−0.0033 (15)
C8	0.071 (2)	0.054 (2)	0.0457 (19)	0.0014 (17)	−0.0218 (16)	−0.0033 (16)
C9	0.064 (2)	0.053 (2)	0.0399 (17)	−0.0030 (17)	−0.0128 (15)	−0.0118 (15)
C10	0.0419 (16)	0.0432 (17)	0.0381 (16)	−0.0067 (14)	−0.0013 (12)	−0.0058 (13)
C11	0.0475 (18)	0.0434 (18)	0.0415 (17)	−0.0024 (14)	−0.0048 (13)	−0.0030 (14)
C12	0.0502 (18)	0.0518 (19)	0.0361 (16)	−0.0011 (15)	−0.0101 (14)	−0.0048 (14)
C13	0.057 (2)	0.057 (2)	0.0455 (18)	−0.0047 (17)	−0.0027 (15)	−0.0164 (15)
C14	0.0508 (18)	0.0411 (18)	0.0532 (19)	0.0047 (15)	−0.0058 (15)	−0.0113 (15)
C15	0.0526 (19)	0.0446 (18)	0.0447 (17)	−0.0018 (15)	−0.0106 (14)	−0.0042 (14)
C16	0.108 (3)	0.062 (2)	0.0320 (17)	−0.002 (2)	−0.0128 (17)	−0.0012 (16)
C17	0.075 (2)	0.056 (2)	0.061 (2)	0.0025 (18)	−0.0060 (17)	−0.0159 (17)
C18	0.0531 (19)	0.0466 (18)	0.0455 (18)	−0.0039 (15)	−0.0170 (15)	−0.0028 (15)
C19	0.0575 (19)	0.0464 (18)	0.0402 (17)	0.0019 (15)	−0.0135 (14)	−0.0064 (14)
C20	0.0535 (18)	0.0468 (18)	0.0393 (17)	−0.0069 (15)	−0.0115 (14)	−0.0055 (14)
C21	0.0447 (17)	0.0410 (17)	0.0409 (16)	−0.0057 (14)	−0.0089 (13)	−0.0063 (13)
C22	0.0467 (17)	0.0448 (18)	0.0368 (16)	−0.0072 (14)	−0.0093 (13)	−0.0029 (13)
C23	0.0511 (18)	0.0489 (18)	0.0361 (16)	−0.0026 (15)	−0.0041 (13)	−0.0074 (14)
C24	0.0471 (18)	0.0418 (18)	0.0463 (18)	0.0014 (14)	−0.0100 (14)	−0.0047 (14)
C25	0.0562 (19)	0.0504 (19)	0.0406 (17)	0.0029 (16)	−0.0095 (14)	0.0032 (14)
C26	0.0533 (18)	0.0527 (19)	0.0328 (15)	−0.0035 (15)	−0.0034 (13)	−0.0051 (14)
C27	0.0429 (17)	0.0414 (17)	0.0444 (17)	−0.0010 (14)	−0.0064 (13)	−0.0066 (13)
C28	0.0535 (19)	0.0431 (17)	0.0476 (18)	0.0018 (15)	−0.0099 (14)	−0.0068 (14)
C29	0.0552 (19)	0.0530 (19)	0.0394 (17)	−0.0035 (16)	−0.0105 (14)	−0.0068 (15)
C30	0.062 (2)	0.060 (2)	0.053 (2)	−0.0035 (17)	−0.0097 (16)	−0.0227 (17)
C31	0.064 (2)	0.0454 (19)	0.068 (2)	0.0114 (17)	−0.0215 (18)	−0.0176 (17)
C32	0.062 (2)	0.0478 (19)	0.0529 (19)	0.0042 (16)	−0.0196 (16)	−0.0022 (16)
C33	0.068 (2)	0.070 (2)	0.0328 (16)	−0.0052 (18)	−0.0047 (15)	0.0020 (15)
C34	0.079 (2)	0.062 (2)	0.058 (2)	0.0128 (19)	−0.0041 (18)	−0.0182 (18)

### Geometric parameters (Å, °)

**Table:**

F1—C12	1.363 (3)	C15—H15	0.9300
F2—C14	1.355 (3)	C16—H16A	0.9600
F3—C29	1.359 (3)	C16—H16B	0.9600
F4—C31	1.349 (3)	C16—H16C	0.9600
O1—C1	1.223 (3)	C17—H17A	0.9600
O2—C5	1.360 (3)	C17—H17B	0.9600
O2—C16	1.428 (3)	C17—H17C	0.9600
O3—C7	1.363 (3)	C18—C19	1.461 (4)
O3—C17	1.432 (3)	C18—C27	1.501 (4)
O4—C18	1.224 (3)	C19—C20	1.324 (4)
O5—C22	1.356 (3)	C19—H19	0.9300
O5—C33	1.425 (3)	C20—C21	1.455 (4)
O6—C24	1.362 (3)	C20—H20	0.9300
O6—C34	1.427 (3)	C21—C26	1.399 (4)
C1—C2	1.465 (4)	C21—C22	1.412 (4)
C1—C10	1.510 (4)	C22—C23	1.388 (4)
C2—C3	1.319 (4)	C23—C24	1.385 (3)
C2—H2	0.9300	C23—H23	0.9300
C3—C4	1.448 (4)	C24—C25	1.384 (4)
C3—H3	0.9300	C25—C26	1.366 (4)
C4—C9	1.398 (4)	C25—H25	0.9300
C4—C5	1.410 (4)	C26—H26	0.9300
C5—C6	1.395 (4)	C27—C32	1.388 (4)
C6—C7	1.391 (4)	C27—C28	1.396 (4)
C6—H6	0.9300	C28—C29	1.358 (4)
C7—C8	1.390 (4)	C28—H28	0.9300
C8—C9	1.364 (4)	C29—C30	1.362 (4)
C8—H8	0.9300	C30—C31	1.365 (4)
C9—H9	0.9300	C30—H30	0.9300
C10—C11	1.387 (4)	C31—C32	1.357 (4)
C10—C15	1.391 (4)	C32—H32	0.9300
C11—C12	1.362 (4)	C33—H33A	0.9600
C11—H11	0.9300	C33—H33B	0.9600
C12—C13	1.366 (4)	C33—H33C	0.9600
C13—C14	1.366 (4)	C34—H34A	0.9600
C13—H13	0.9300	C34—H34B	0.9600
C14—C15	1.358 (4)	C34—H34C	0.9600
			
C5—O2—C16	119.8 (2)	H17A—C17—H17C	109.5
C7—O3—C17	118.9 (2)	H17B—C17—H17C	109.5
C22—O5—C33	119.3 (2)	O4—C18—C19	121.0 (3)
C24—O6—C34	119.0 (2)	O4—C18—C27	118.9 (3)
O1—C1—C2	121.2 (3)	C19—C18—C27	120.1 (2)
O1—C1—C10	119.4 (3)	C20—C19—C18	121.5 (3)
C2—C1—C10	119.4 (2)	C20—C19—H19	119.2
C3—C2—C1	122.7 (3)	C18—C19—H19	119.2
C3—C2—H2	118.6	C19—C20—C21	127.9 (3)
C1—C2—H2	118.6	C19—C20—H20	116.0
C2—C3—C4	128.0 (3)	C21—C20—H20	116.0
C2—C3—H3	116.0	C26—C21—C22	117.2 (2)
C4—C3—H3	116.0	C26—C21—C20	122.7 (3)
C9—C4—C5	116.9 (3)	C22—C21—C20	120.2 (3)
C9—C4—C3	122.9 (3)	O5—C22—C23	123.3 (3)
C5—C4—C3	120.2 (2)	O5—C22—C21	115.6 (2)
O2—C5—C6	122.7 (3)	C23—C22—C21	121.0 (3)
O2—C5—C4	115.8 (2)	C24—C23—C22	119.3 (3)
C6—C5—C4	121.6 (3)	C24—C23—H23	120.3
C7—C6—C5	118.8 (3)	C22—C23—H23	120.3
C7—C6—H6	120.6	O6—C24—C25	115.0 (2)
C5—C6—H6	120.6	O6—C24—C23	124.3 (3)
O3—C7—C8	115.4 (3)	C25—C24—C23	120.7 (3)
O3—C7—C6	124.1 (3)	C26—C25—C24	119.7 (3)
C8—C7—C6	120.5 (3)	C26—C25—H25	120.2
C9—C8—C7	119.8 (3)	C24—C25—H25	120.2
C9—C8—H8	120.1	C25—C26—C21	122.1 (3)
C7—C8—H8	120.1	C25—C26—H26	118.9
C8—C9—C4	122.4 (3)	C21—C26—H26	118.9
C8—C9—H9	118.8	C32—C27—C28	119.0 (3)
C4—C9—H9	118.8	C32—C27—C18	118.4 (3)
C11—C10—C15	119.6 (3)	C28—C27—C18	122.5 (3)
C11—C10—C1	122.6 (3)	C29—C28—C27	118.5 (3)
C15—C10—C1	117.8 (2)	C29—C28—H28	120.7
C12—C11—C10	118.3 (3)	C27—C28—H28	120.7
C12—C11—H11	120.9	C28—C29—F3	118.5 (3)
C10—C11—H11	120.9	C28—C29—C30	123.5 (3)
C11—C12—F1	118.5 (3)	F3—C29—C30	118.0 (3)
C11—C12—C13	123.9 (3)	C29—C30—C31	116.6 (3)
F1—C12—C13	117.6 (3)	C29—C30—H30	121.7
C12—C13—C14	116.0 (3)	C31—C30—H30	121.7
C12—C13—H13	122.0	F4—C31—C32	118.7 (3)
C14—C13—H13	122.0	F4—C31—C30	118.1 (3)
F2—C14—C15	118.3 (3)	C32—C31—C30	123.2 (3)
F2—C14—C13	118.0 (3)	C31—C32—C27	119.1 (3)
C15—C14—C13	123.7 (3)	C31—C32—H32	120.5
C14—C15—C10	118.5 (3)	C27—C32—H32	120.5
C14—C15—H15	120.8	O5—C33—H33A	109.5
C10—C15—H15	120.8	O5—C33—H33B	109.5
O2—C16—H16A	109.5	H33A—C33—H33B	109.5
O2—C16—H16B	109.5	O5—C33—H33C	109.5
H16A—C16—H16B	109.5	H33A—C33—H33C	109.5
O2—C16—H16C	109.5	H33B—C33—H33C	109.5
H16A—C16—H16C	109.5	O6—C34—H34A	109.5
H16B—C16—H16C	109.5	O6—C34—H34B	109.5
O3—C17—H17A	109.5	H34A—C34—H34B	109.5
O3—C17—H17B	109.5	O6—C34—H34C	109.5
H17A—C17—H17B	109.5	H34A—C34—H34C	109.5
O3—C17—H17C	109.5	H34B—C34—H34C	109.5
			
O1—C1—C2—C3	0.5 (5)	O4—C18—C19—C20	12.7 (5)
C10—C1—C2—C3	179.9 (3)	C27—C18—C19—C20	−165.8 (3)
C1—C2—C3—C4	179.1 (3)	C18—C19—C20—C21	−179.8 (3)
C2—C3—C4—C9	2.3 (5)	C19—C20—C21—C26	8.0 (5)
C2—C3—C4—C5	−178.4 (3)	C19—C20—C21—C22	−172.6 (3)
C16—O2—C5—C6	−6.5 (4)	C33—O5—C22—C23	2.4 (4)
C16—O2—C5—C4	174.0 (3)	C33—O5—C22—C21	−178.5 (2)
C9—C4—C5—O2	178.1 (3)	C26—C21—C22—O5	−178.5 (2)
C3—C4—C5—O2	−1.2 (4)	C20—C21—C22—O5	2.0 (4)
C9—C4—C5—C6	−1.4 (4)	C26—C21—C22—C23	0.6 (4)
C3—C4—C5—C6	179.3 (3)	C20—C21—C22—C23	−178.8 (3)
O2—C5—C6—C7	−179.4 (3)	O5—C22—C23—C24	178.5 (2)
C4—C5—C6—C7	0.1 (4)	C21—C22—C23—C24	−0.6 (4)
C17—O3—C7—C8	175.6 (3)	C34—O6—C24—C25	−178.2 (3)
C17—O3—C7—C6	−4.4 (4)	C34—O6—C24—C23	0.5 (4)
C5—C6—C7—O3	−179.3 (3)	C22—C23—C24—O6	−178.2 (3)
C5—C6—C7—C8	0.7 (4)	C22—C23—C24—C25	0.5 (4)
O3—C7—C8—C9	180.0 (3)	O6—C24—C25—C26	178.5 (3)
C6—C7—C8—C9	0.0 (5)	C23—C24—C25—C26	−0.3 (4)
C7—C8—C9—C4	−1.5 (5)	C24—C25—C26—C21	0.4 (5)
C5—C4—C9—C8	2.1 (4)	C22—C21—C26—C25	−0.5 (4)
C3—C4—C9—C8	−178.6 (3)	C20—C21—C26—C25	178.9 (3)
O1—C1—C10—C11	−175.4 (3)	O4—C18—C27—C32	−1.7 (4)
C2—C1—C10—C11	5.2 (4)	C19—C18—C27—C32	176.7 (3)
O1—C1—C10—C15	5.4 (4)	O4—C18—C27—C28	−178.6 (3)
C2—C1—C10—C15	−174.0 (3)	C19—C18—C27—C28	−0.1 (4)
C15—C10—C11—C12	−0.3 (4)	C32—C27—C28—C29	0.6 (4)
C1—C10—C11—C12	−179.6 (3)	C18—C27—C28—C29	177.4 (3)
C10—C11—C12—F1	−177.0 (2)	C27—C28—C29—F3	−179.8 (3)
C10—C11—C12—C13	0.6 (5)	C27—C28—C29—C30	0.8 (5)
C11—C12—C13—C14	0.3 (5)	C28—C29—C30—C31	−1.7 (5)
F1—C12—C13—C14	177.9 (3)	F3—C29—C30—C31	178.9 (3)
C12—C13—C14—F2	178.3 (3)	C29—C30—C31—F4	−179.2 (3)
C12—C13—C14—C15	−1.5 (5)	C29—C30—C31—C32	1.2 (5)
F2—C14—C15—C10	−178.1 (3)	F4—C31—C32—C27	−179.5 (3)
C13—C14—C15—C10	1.7 (5)	C30—C31—C32—C27	0.1 (5)
C11—C10—C15—C14	−0.7 (4)	C28—C27—C32—C31	−1.0 (5)
C1—C10—C15—C14	178.6 (3)	C18—C27—C32—C31	−177.9 (3)

### Hydrogen-bond geometry (Å, °)

**Table:**

*D*—H···*A*	*D*—H	H···*A*	*D*···*A*	*D*—H···*A*
C25—H25···F4^i^	0.93	2.59	3.375 (3)	142
C13—H13···F4^ii^	0.93	2.46	3.303 (4)	151
C9—H9···F3^iii^	0.93	2.66	3.532 (3)	156
C30—H30···F2^ii^	0.93	2.47	3.303 (4)	149
C8—H8···F2^i^	0.93	2.46	3.369 (3)	166
C28—H28···F1^iii^	0.93	2.53	3.437 (3)	166

Symmetry codes: (i) *x*+1, *y*−1, *z*; (ii) −*x*−1, −*y*+2, −*z*+2; (iii) −*x*, −*y*+1, −*z*+2.
